# Protein phosphatase 2A modulates podocyte maturation and glomerular functional integrity in mice

**DOI:** 10.1186/s12964-019-0402-y

**Published:** 2019-08-06

**Authors:** Xiujuan Zhu, Yuhong Ye, Chengxian Xu, Cunji Gao, Yingying Zhang, Jing Zhou, Weiqiang Lin, Jianhua Mao

**Affiliations:** 1 Department of Nephrology, The Children Hospital of Zhejiang University School of Medicine, #57 Zhugan Lane, Hangzhou, 310003 Zhejiang Province People’s Republic of China; 2 Chronic Disease Research Institute, Department of Nutrition and Food Hygiene, Zhejiang University School of Public Health, Hangzhou, 310058 Zhejiang Province People’s Republic of China; 3 Harvard Center for Polycystic Kidney Disease Research and Renal Division, Department of Medicine, Brigham and Women’s Hospital and Harvard Medical School, Boston, MA02115 USA; 4 Institute of Translational Medicine, School of Medicine, Zhejiang University, Hangzhou, 310058 Zhejiang Province People’s Republic of China

**Keywords:** PP2A, Dephosphorylation, Podocyte, Proteinuria, Glomerulopathy, YB-1

## Abstract

**Background:**

Protein phosphorylation & dephosphorylation are ubiquitous cellular processes that allow for the nuanced and reversible regulation of protein activity. Protein phosphatase 2A (PP2A) is a multifunction phosphatase that is well expressed in all cell types of kidney during early renal development, though its functions in kidney remains to be elucidated.

**Methods:**

PP2A conditional knock-out mice was generated with PP2A fl/fl mice that were crossed with Podocin-Cre mice. The phenotype of Pod-PP2A–KO mice (homozygous for the floxed PP2A allele with Podocin-Cre) and littermate PP2A fl/fl controls (homozygous for the PP2A allele but lacking Podocin-Cre) were further studied. Primary podocytes isolated from the Pod-PP2A-KO mice were cultured and they were then employed with sing label-free nano-LC − MS/MS technology on a Q-exactive followed by SIEVE processing to identify possible target molecular entities for the dephosphorylation effect of PP2A, in which Western blot and immunofluorescent staining were used to analyze further.

**Results:**

Pod-PP2A–KO mice were developed with weight loss, growth retardation, proteinuria, glomerulopathy and foot process effacement, together with reduced expression of some slit diaphragm molecules and cytoskeleton rearrangement of podocytes. Y box protein 1 (YB-1) was identified to be the target molecule for dephosphorylation effect of PP2A. Furthermore, YB-1 phosphorylation was up-regulated in the Pod-PP2A–KO mice in contrast to the wild type controls, while total and un-phosphorylated YB-1 both was moderately down-regulated in podocytes from the Pod-PP2A-KO mice.

**Conclusion:**

Our study revealed the important role of PP2A in regulating the development of foot processes and fully differentiated podocytes whereas fine-tuning of YB-1 via a post-translational modification by PP2A regulating its activity might be crucial for the functional integrity of podocytes and glomerular filtration barrier.

**Graphic abstract:**

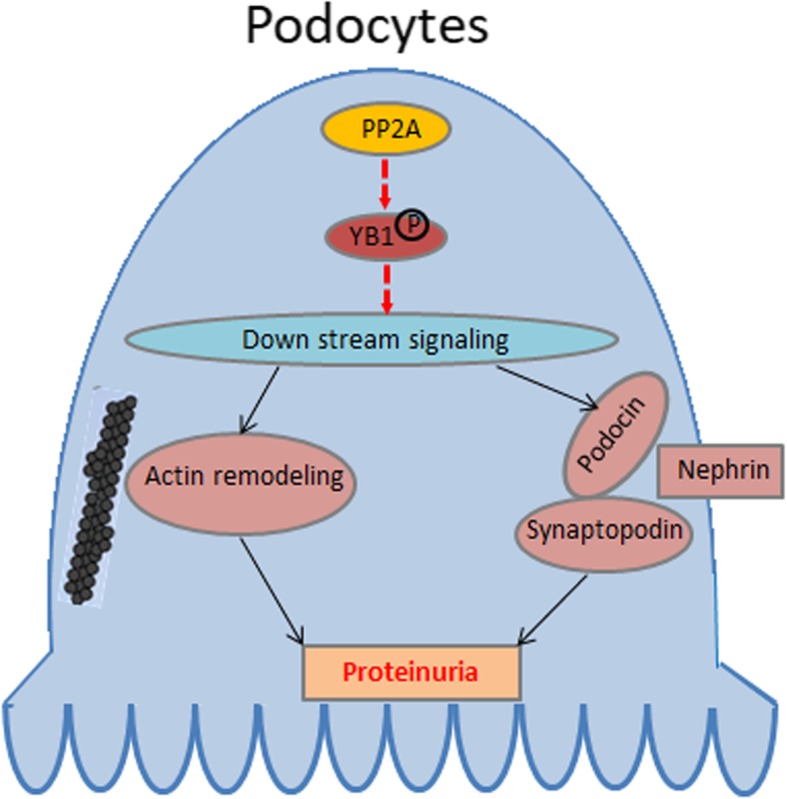

**Electronic supplementary material:**

The online version of this article (10.1186/s12964-019-0402-y) contains supplementary material, which is available to authorized users.

## Background

Chronic kidney disease is a prevalent condition that affects millions of people worldwide and is a major risk factor of morbidity and mortality. One of the most important clinical risk factor in the progression of kidney disease is proteinuria [1]. Podocytes are well-differentiated cells that together with the glomerular basement membrane and the adjacent fenestrated endothelial cell form the glomerular filtration barrier. Podocytes exhibit a unique cytoskeletal architecture that is fundamentally linked to their function in maintaining kidney filtration barrier [2]. Recently, many molecules such as Nephrin [3], CD2AP [4], Podocin [5, 6] and TRPC6 [7] have been identified to play a key role in keeping the functional integrity of glomerular filtration and preventing plasma protein leakage to urine.

Protein phosphorylation & dephosphorylation are ubiquitous cellular processes that allow for the nuanced and reversible regulation of protein activity. Protein phosphatase 2A (PP2A) is a multifunction phosphatase that is ubiquitously expressed in eukaryotic cells which consists of a complex with three subunits including a scaffold subunit A, a catalytic subunit C, and a regulatory subunit B [8–10]. PP2A was suggested to regulate various cellular processes such as signal transduction, cytoskeleton dynamics, promoting cell transformation [11], and angiogenesis [12, 13].

Svennilson et al. [14] reported that the mRNA of PP2A is well expressed in all cell types during early kidney development, and low doses of okadaic acid (an exogenous inhibitor of PP2A) inhibited the growth of the early (E13) embryonic kidney and caused disturbances of nephron formation in E15 kidneys. Everett et al. [15] demonstrated that the catalytic subunit protein of PP2A (PP2Ac) particularly concentrated in the podocytes of glomeruli in the newborn kidney. Kobayashi et al. [16] reported that inhibition of PP2A with okadaic acid in a conditionally immortalized mouse podocyte cell line suppressed microtubule elongation, abolished process formation. Kumar et al. [9] revealed selective inhibition of PP2A restored insulin induced phosphorylation of AKT, FOXO1, SIRT1 activity, p53 degradation, and podocyte death. These previous studies implied that the important role of PP2A in maintaining cytoskeleton development, podocyte survival, nephron growth and differentiation in the kidney.

In the present study, we report functional, morphologic, and molecular alterations induced by conditional inactivation of PP2A selectively in podocytes of mice. The mutant mice manifest a sequelae of podocyte and glomerular abnormalities, which included weight loss, growth retardation, proteinuria, renal dysplasia, glomerulopathy and foot process (FP) effacement, together with reduced expression of slit diaphragm molecule and cytoskeleton rearrangement of podocytes.

## Materials and methods

### Human renal biopsy samples

Specimens of normal kidney tissue were obtained from an intact pole of kidney removed for a circumscribed tumor and aspirated renal biopsy tissues from isolated hematuria patients, and without evidence of mesangial proliferation, podocyte process effacement or alteration in glomerular basement membrane under electromicroscope (EM). This study was conducted according to the principles of the Declaration of Helsinki and was approved by the Research Ethics Committee of The Children Hospital of Zhejiang University School of Medicine (20151010) after informed written consent was obtained from the patients.

### Animal treatment protocols

Studies were performed on 8-week-old male and female BALB/c mice obtained from Charles River Lab and cultured in the laboratory Animal Center of Zhejiang University according to SPF standard. The mice were randomly divided into 2 groups: (1) control mice (*n* = 10, 5 male and 5 female, respectively); (2) ADM treatment (*n* = 10, 5 male and 5 female, respectively), ADM Mice were administered intravenously by tail vein injection with ADM (Sigma) (10 mg/kg) to induce glomerular injury, and they were then sacrificed 2 (5 mice) or 4 weeks (5 mice) after the injection.

### Creation and genotyping of podocyte-specific PP2A-KO mice

To selectively delete PP2A in glomerular podocytes, PP2A fl/fl mice [17] were crossed with Podocin-Cre mice [18] to generate a podocyte-specific knockout of PP2A (PP2A–KO). Tail genotyping was performed by PCR according to protocols as previously described [17, 18]. PP2A–KO mice (homozygous for the floxed PP2A allele with Podocin-Cre) and littermate PP2A fl/fl controls (homozygous for the PP2A allele but lacking Podocin-Cre) were used in the experiments.

### Urine albumin and creatinine measurements

Urine samples were collected from the Pod-PP2A–KO and controls. Albuminuria was qualitatively assessed by 10% SDS-PAGE followed by Coomassie blue staining [19]. Urine albumin levels were measured in duplicate using an albumin ELISA quantitation kit according to the manufacturer’s protocol (Millipore, Michigan, USA), and the absorbance read at 450 nm (Bio-Rad Microplate Reader, San Jose, USA) as previously described [19]. Urine and plasma creatinine were measured in duplicate for each sample with an ELISA quantitation kit (Cayman, Michigan, USA) at an absorbance of 490 nm (Bio-Rad Microplate Reader, San Jose, USA).

### Pathology and immunofluorescent staining of kidney tissues in mice

After anesthetized with chloral hydrate and then sacrificed, kidneys were removed and fixed with 4% paraformaldehyde (PFA) overnight, and fixed sections stored in 20% sucrose overnight and then immersed in O.C.T Compound (Tissue-Tek) were further processed for HE & PAS staining. For immunofluorescent staining, kidney sections from OCT in both human and mice were blocked with 3% BSA for 1 h at room temperature (RT). Then incubated with the appropriate primary Abs overnight at 4 °C followed by incubation with Alexa Fluor 488 and/or 594–conjugated secondary Abs (Life technology) for 1 h at RT. Images were took by Nikon A1 Ti laser scanning confocal microscope. For transmission electromicroscope, kidneys were post-fixed with Palade’s osmium. EM was performed by the Center of electron Microscopy, Zhejiang University School of Medicine. For scanning EM, kidneys were post-fixed with osmium in 0.1 M sodium cacodylate and 0.1 M sucrose and performed by Analysis Center of Agrobiology and Environmental Sciences& Institute of Agrobiology and Environmental Sciences, Zhejiang University.

### Antibodies

Antibodies used in this study were as follows: Goat anti-synaptopodin (Santa Cruz), Rabbit anti-YB-1 (Abcam), Rabbit anti-PP2A (Abcam), Alexa Fluor 488 donkey anti-rabbit IgG (Life Technologies), Alexa Fluor 594 donkey anti-mouse IgG (Life Technologies), BMP7 (Abcam), a-SAM (Abcam), Desmin (Proteintech), ZO-1 (Proteintech), WT-1 (Abcam), Nephrin (R&D Systems), Podocin (Abcam).

### Cell culture

Isolation of glomeruli from mouse was performed as described previously [20], then plated on dishes coated with collagen at 37 °C in RPMI 1640 medium (Hyclone) with 10% FBS (GIBCO), 100 U/ml penicillin and 100 μg/ml streptomycin. We detach podocyte from other glomerular cells after digestion with 0.25% trypsin-EDTA (GIBCO) after about 7–8 days, followed by sieving through a 40-μm cell strainer (Falcon; BD Biosciences), and then culture on collagen typIcoated dishes. Podocytes of passages 1 or 2 were used in all experiments. To study the role of the cytoskeleton, podocytes were cultured with cytochalasin D (20 μg/ml) for 30 min.

The conditionally immortalized mouse podocyte cell line (MPC5) used in this study was kindly provided by Dr. Peter Mundel (Mount Sinai School of Medicine, New York, NY). To propagate podocytes, cells were cultured at 33 °C in RPMI 1640 medium supplemented with 10% fetal bovine serum (FBS) and 10 U/ml mouse recombinant interferon-γ (R&D Systems, Minneapolis, MN) to increase the expression of a thermosensitive T antigen. To induce differentiation, podocytes were grown under nonpermissive conditions at 37 °C in the absence of interferon-γ for 14 days. Cultural protocols were as previously described [21–23].

### Western blot

Cells were lysed into RIPA (Themo fisher) with protease inhibitor cocktail (Roche Diagnostics) and PhosSTOP (Roche). Protein concentrations were measured by the Bio-Rad Protein Assay and then denatured for 5 min at 96 °C, resolved 6–12% gradient SDS-PAGE gels, and transferred to the PVDF membranes (Millipore). The membrane was blocked with 3% BSA in 1× TBST and then incubated with the appropriate primary Ab at 4 °C overnight. Following 3 washes with 1× TBST, the appropriate HRP-labeled anti-IgG secondary Ab (Jakson) was added and signals was detected using enhanced chemiluminescence reagents (Themo fisher). Phos-tag Acrylamide were used as previously [24] to evaluate protein phosphorylation.

### MS/MS protein identification and quantification

For label-free, relative, quantitative analysis, we collected podocytes from the control and podocyte-specific PP2A-knockout mice at 4 weeks of age, and then they were then analyzed by the Ptm-biolab in Hangzhou to detect the phosphorylation as described [24]. After trypsin digestion, peptide was reconstituted in 0.5 M TEAB and processed according to the manufacturer’s protocol for 6-plex TMT kit. The sample was then fractionated into fractions by high pH reverse-phase HPLC using Agilent 300Extend C18 column, Peptide mixtures were first incubated with IMAC microspheres suspension with vibration. The IMAC microspheres with enriched phosphopeptides were collected by centrifugation, and the supernatant was removed. To remove nonspecifically adsorbed peptides, the IMAC microspheres were washed with 50% ACN/6% TFA and 30% ACN/0.1% TFA, sequentially. To elute the enriched phosphopeptides from the IMAC microspheres, elution buffer containing 10% NH4OH was added and the enriched phosphopeptides were eluted with vibration. The supernatant containing phosphopeptides was collected and lyophilized for LC-MS/MS analysis. The resulting MS/MS data was processed using MaxQuant with integrated Andromeda search engine (v.1.5.1.8). Tandem mass spectra were searched against *SwissProt* Mouse database concatenated with reverse decoy database.

### Statistics

Results are presented as mean ± SEM. Statistical analysis was performed using 2-tailed Student’s t test). A *P* < 0.05 was considered to be statistically significant.

## Results

### PP2A expression in human and mouse kidney

**Fig. 1:**
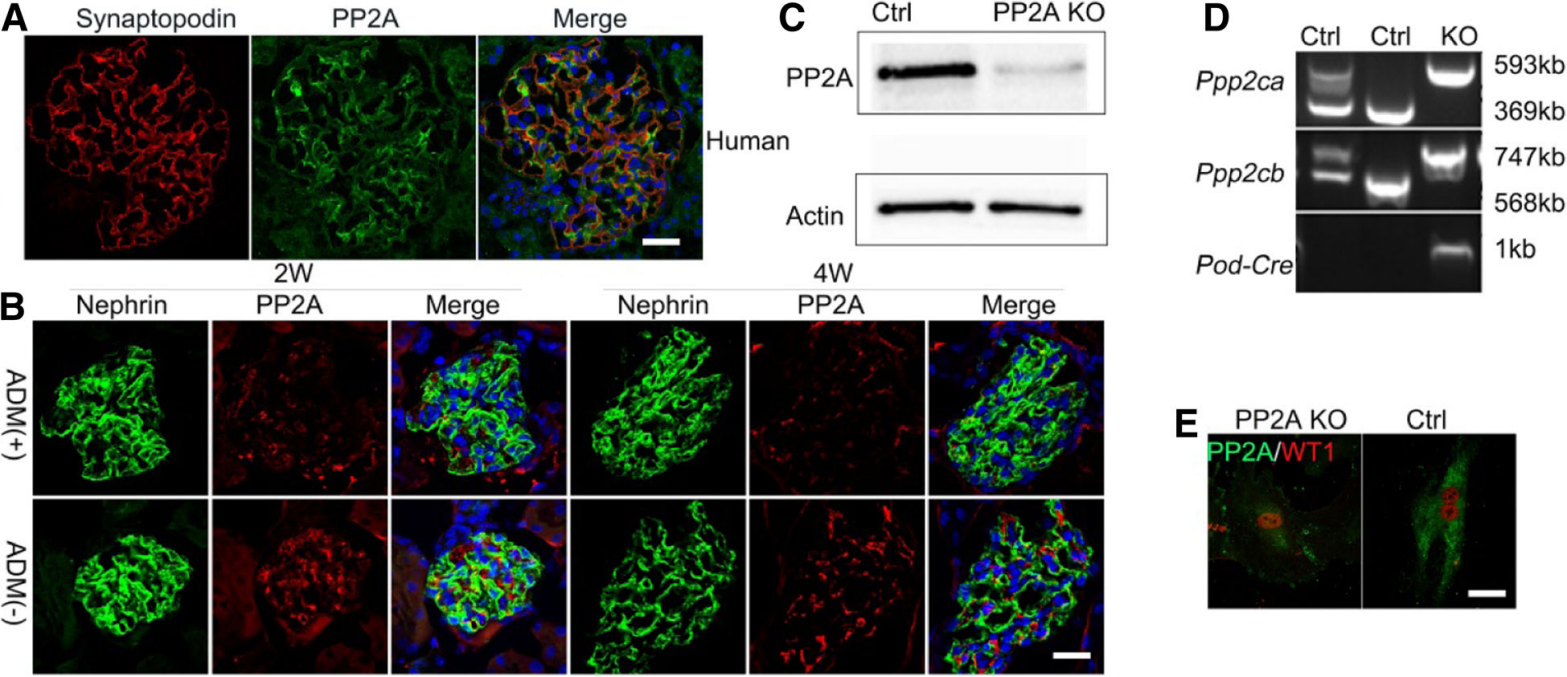
The expression of PP2A in human and ADM nephropathy animal models and Generation of podocyte-specific pp2a knock out mice. **a** Double immunofluorescence demonstrates PP2A expression in the podocyte of normal human kidney. Scale bars: 50 μm. **b** The expression of PP2A in the podocytes from ADM nephropathy model in the BALB/c mice (*n* = 5 per time point). Scale bars: 50 μm. **c** The results of Western blotting indicate the PP2A expression in purified control (Ctrl) podocytes and lack of PP2A expression in podocytes harvest from the PP2A specific-knock out mice. **d** Identification of pp2a and Podocin-Cre DNA level by tail genotyping (4 week). **e** Primary podocytes isolated from Pod-PP2A-KO mice were plated on collagen type I–coated glass coverslips and then stained for WT1 (Red) and PP2A (Green). Compared with the control, the expression of PP2A was also down-regulated in primary podocytes from Pod-PP2A-KO mice. Scale bars: 25 μm

PP2A in the human renal glomeruli was shown in Fig. 1a. PP2A showed a podocyte−like staining pattern in normal human glomeruli as synaptopodin. Further, the expression of PP2A was decreased in the ADM nephropathy model, compared with control BALB/c mice which received normal saline injection only (Fig. 1b).

### Podocyte-specific PP2A-KO mice

Global loss of the PP2A gene is embryonic lethal [17, 25], so, to study the function of PP2A in podocyte of kidney, podocyte-specific PP2A-KO mice was generated by the Cre-Loxp system based on podocin-Cre, which can express Cre recombinase at E13–E14 [18]. Compound PP2A heterozygote mice were mated to generate podocyte-specific PP2A-KO mice (Pod-PP2A–KO). Pod-PP2A–KO, PP2A fl/fl mice (used as controls) were born in the same Mendelian ratio as identified by tail genotyping (Fig. 1d). PP2A loss in podocyte isolated from Pod-PP2A–KO mice was confirmed by Western blot (Fig. 1c), by tail genotyping (Fig. 1d) and by immunofluorescences (Fig. 1e).

### Pod-PP2A–KO mice developed weight loss, growth retardation, proteinuria and lethargy

**Fig. 2:**
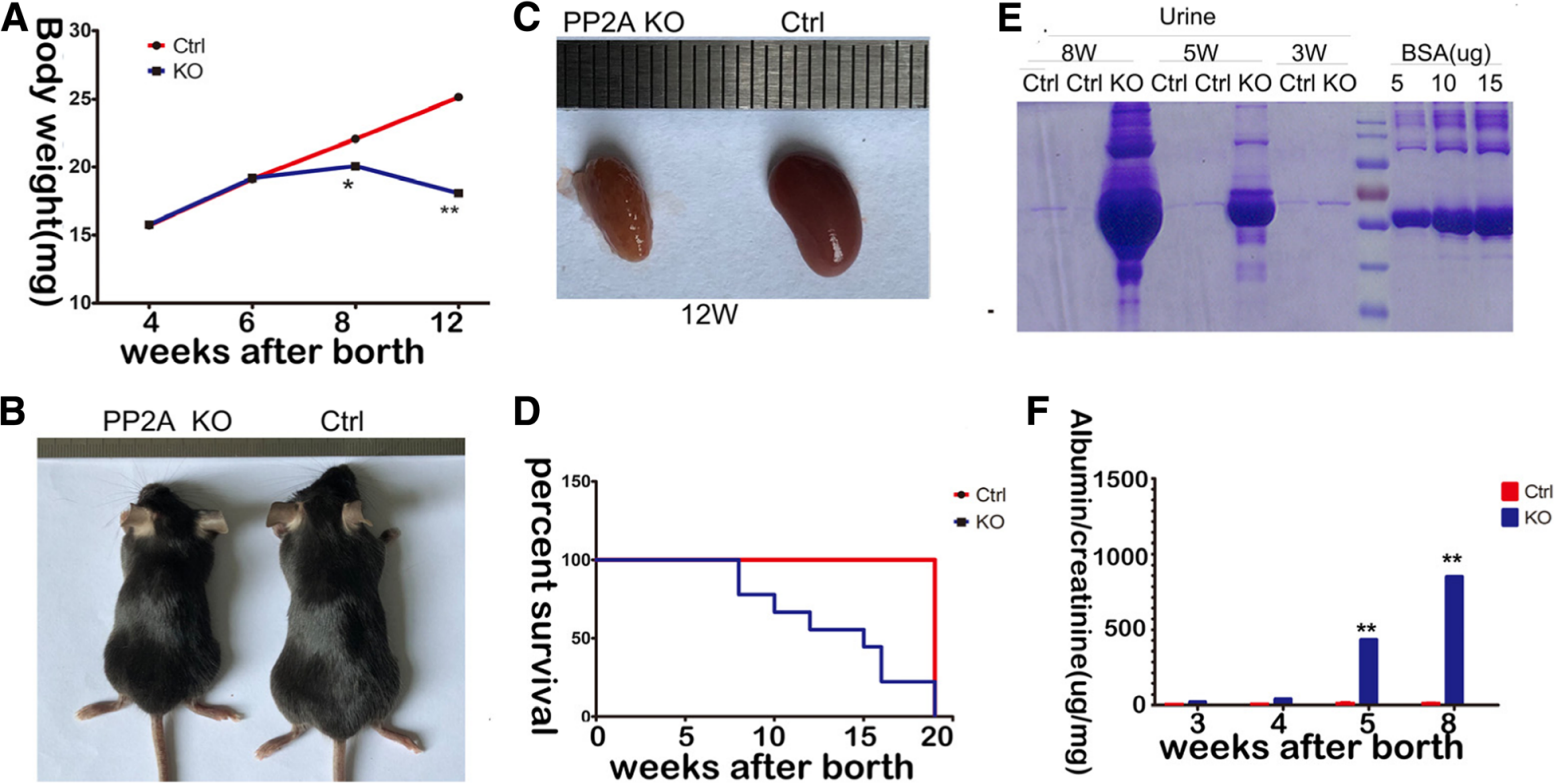
Podocyte-specific loss of PP2A leads to proteinuria and renal dysplasia. **a** Compared with the control, the Pod-PP2A-KO mice fail to gain weight at 6 weeks of age. *n* = 10 mice.* *P* < 0.05, **P < 0.001. **b** Representative images of the control mice and Pod-PP2A-KO mice at 12 weeks of age. **c** Representative images of kidney from control and Pod-PP2A-KO mice at 12 weeks of age. The kidney from the Pod-PP2A-KO mice was smaller than that of control, even after the size of kidney was balanced by the size of body. **d** Survival curve of Pod-PP2A-KO mice demonstrates greater than 50% death at 12 weeks of age. n = 10 mice. **e** SDS-PAGE (Coomassie blue staining) of urine from Pod-PP2A-KO mice, demonstrating albuminuria at 3, 5 and 8 weeks of age. 2 μl of standard BSA and urine were loaded in each lane. **f** Quantification of urinary albumin normalized to creatinine at 3, 4, 6, and 8 weeks of age. *n* = 8 mice

Pod-PP2A–KO mice appeared normal at birth, but their weight gain began to fall below than that of PP2A fl/fl mice at 6 weeks of age (Fig. 2a). At 12 weeks of age, the animals were smaller, growth retardation and severely lethargic (Fig. 2b). The kidney harvested from the Pod-PP2A–KO mice showed a shrunken appearance at 12 weeks when compared to PP2A fl/fl mice (Fig. 2c). More than 70% mice die at 15 weeks of age (Fig. 2d). Urine collected from the Pod-PP2A–KO mice demonstrated albuminuria beginning as early as week 4th and continuing to progress by Coomassie-blue–stained SDS-PAGE gels (Fig. 2e) as described [19], then validated by ELISA (Fig. 2f).

### Deletion of PP2A in podocytes results in glomerulopathy and foot process effacement

**Fig. 3:**
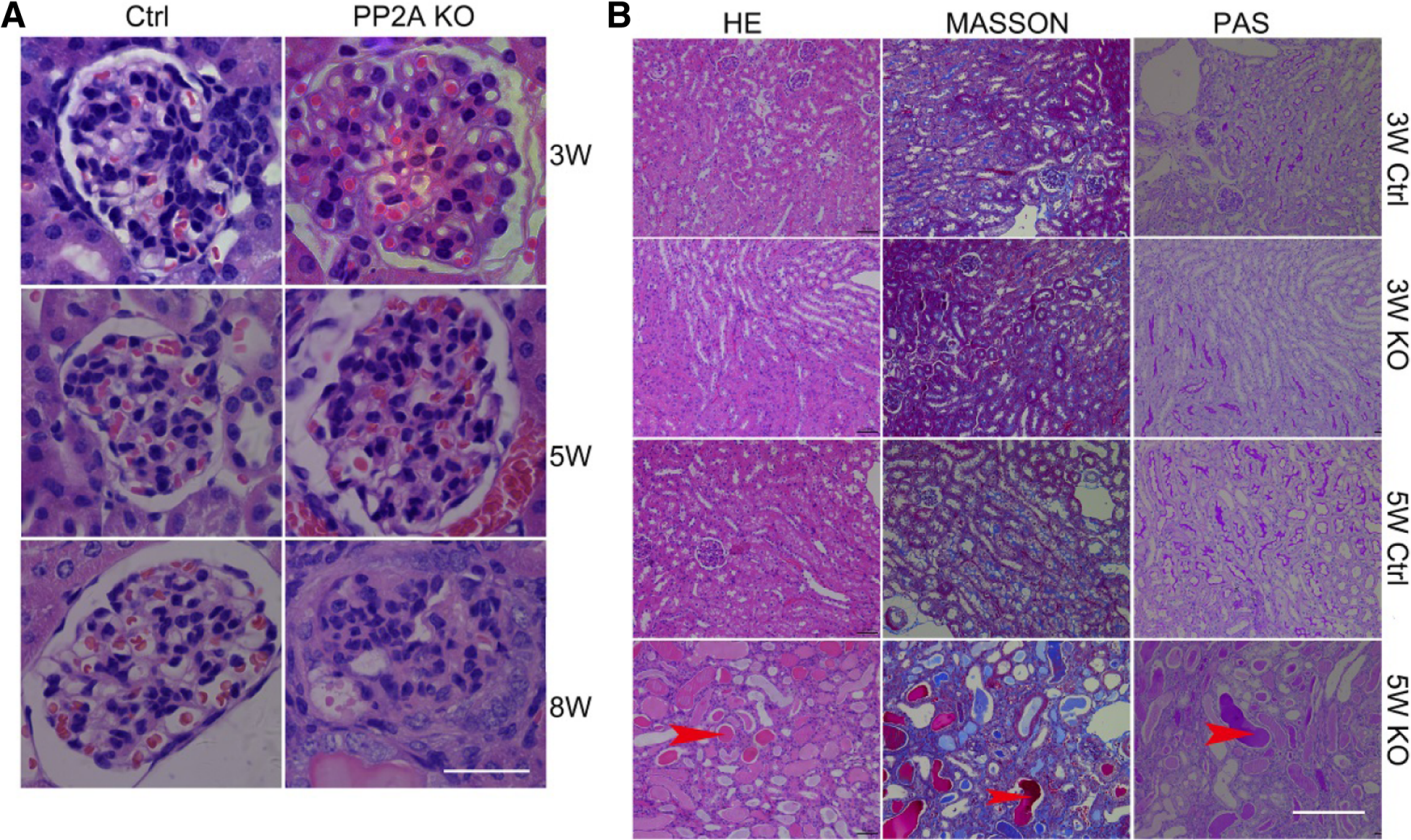
Podocyte-specific loss of PP2A results in progressive glomerulosclerosis and interstitial fibrosis. **a** Representative HE images of glomeruli from Pod-PP2A-KO mice revealing histological evidence of dilated glomerular capillary loops at 5 weeks of age (arrowheads), which progresses to diffuse glomerulosclerosis by 8 weeks. Scale bar: 50 μm. **b** Proteinaceous casts (arrowhead) and severe interstitial fibrosis are observed in kidney section from Pod-PP2A-KO mice at 8 weeks of age by the HE, Masson, and PAS staining. Scale bar: 50 μm

**Fig. 4:**
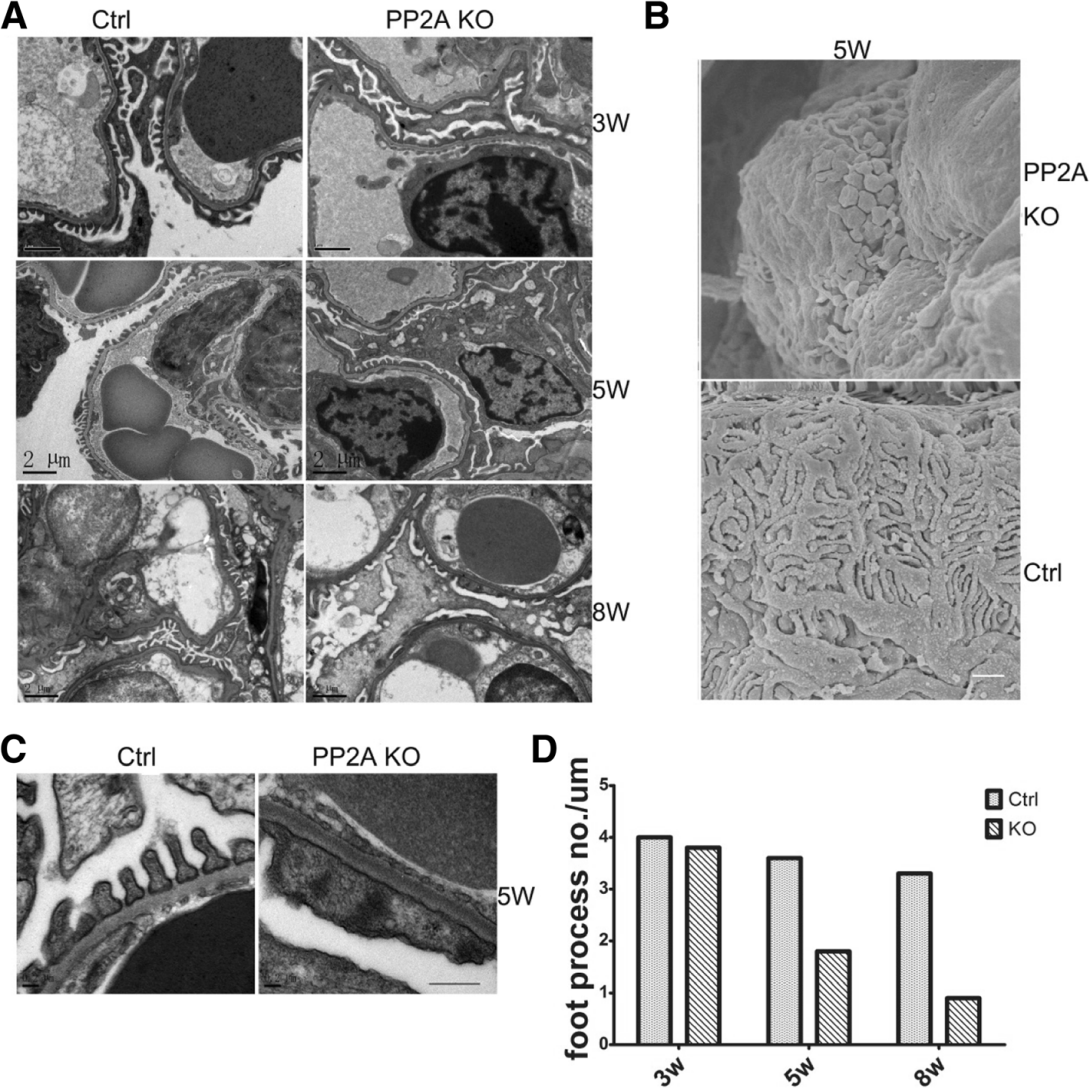
Podocyte-specific knockout of PP2A results in foot process effacement. **a** Transmission electron microscope illustrates foot process effacement at 3 weeks, 5 weeks and 8 weeks of age. Scale bar: 2 μm. **b** Scanning electron microscope illustrates the dramatic loss of podocyte foot process interdigitation and destruction of the major processes in Pod-PP2A-KO mice at 5 weeks of age. Scale bars: 2 μm. **c** High-magnification micrographs shows foot process effacement in glomeruli from Pod-PP2A-KO mice at 5 weeks of age. Scale bars: 1 μm. **d** Quantification of the number of podocyte foot processes per μm of glomerular basement membrane in Pod-PP2A-KO mice at 3 weeks, 5 weeks and 8 weeks of age. *n* = 2 mice at each time point

Histological examination of Pod-PP2A–KO kidneys revealed normal features at birth. However, at 5-weeks of age, approximately 25% of glomeruli revealed severe glomerular capillary dilatation. At 8 weeks of age, the glomeruli had undergone a process of global sclerosis (Fig. 3a) and the kidney further demonstrated severe interstitial fibrosis, tubular dilatation, and proteinaceous casts (Fig. 3b). In-depth understanding the ultrastructural characteristics of the glomerulus, both scanning and transmission EM were performed for the section of Pod-PP2A–KO and control kidneys. In Pod-PP2A–KO mice at 5-weeks after birth, the dramatic loss of podocyte foot process interdigitation and destruction of the major processes could be observed by scanning EM (Fig. 4a). At postnatal week 5th, there was significant foot process effacement (Fig. 4b-d), in line with our findings observed from the scanning EM.

### Loss of podocyte PP2A results in a modest reduction of expression of slit diaphragm molecule

**Fig. 5:**
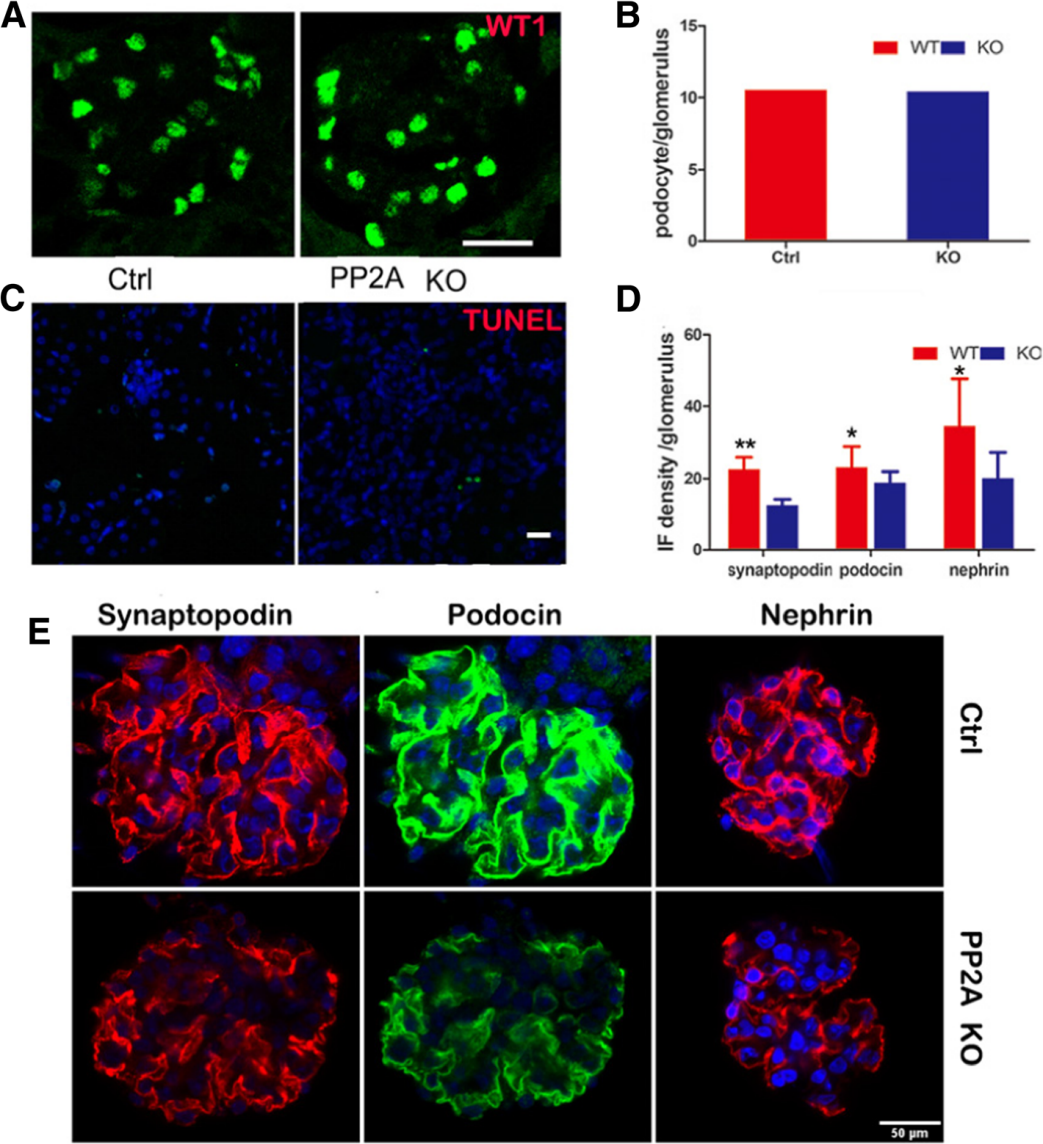
Loss of podocyte PP2A results in a modest reduction of the expression of some epithelial cell markers. **a** Expression of WT1 in glomeruli from Pod-PP2A-KO mice and control at 5 weeks of age. Scale bar: 50 μm. **b** Quantification of WT1 staining in podocytes per glomerulus from **a**. *n* = 6 experiments. No significant loss of WT1 expression was seen between the podocytes from Pod-PP2A-KO mice and control. **c** The results of Tunel assay in glomerulus and tubules demonstrate that there is no apoptosis in the PP2A knockout mice. **d** Quantification of immunofluorescence density for synaptopodin, podocin and nephrin per glomerulus, *n* = 6 experiments. **e** The expression of synaptopodin, podocin and nephrin in glomeruli by immunofluorescence and confocal microscopic from Pod-PP2A-KO mice and control. Scale bar: 50 μm

To demonstrate whether podocyte loss might occur in the Pod-PP2A–KO mice as a result of cell detachment or not, WT1 staining was used to measure the number of podocyte in glomeruli as WT1 is the podocyte-specific marker. Compared with control mice, kidney from Pod-PP2A–KO mice at 4 weeks of age revealed similar podocyte numbers (Fig. 5a; quantified in Fig. 5b), indicating that no significant podocyte loss occurred in the Pod-PP2A–KO mice. Furthermore, TUNEL staining demonstrated no evidence of apoptosis within the glomeruli from Pod-PP2A–KO mice (Fig. 5c). The expression of silt diagram molecule which is critical for the integrality of the glomerular filtration barrier was investigated in present study. The expression of slit diaphragm molecule such as synaptopodin, nephrin and podocin, were analyzed by immunofluorescences, and the expression of synaptopodin, nephrin and podocin was down regulated within the glomeruli from Pod-PP2A–KO mice when compared to the control (Fig. 5e) and quantified in (Fig. 5d).

### Deletion of PP2A induced cytoskeleton rearrangement of podocytes

**Fig. 6:**
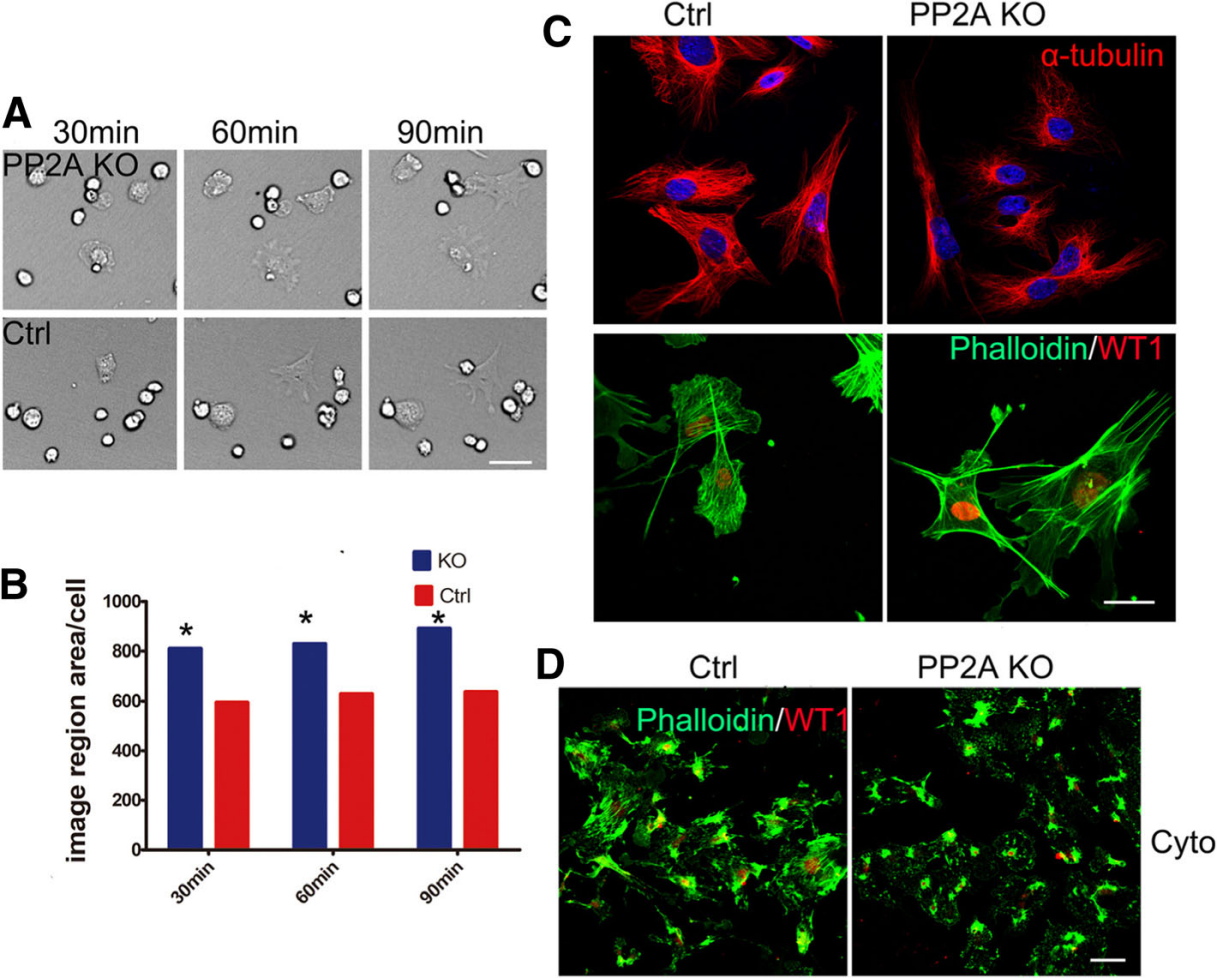
Cell spreading and cytoskeleton rearrangement in podocyte from Pod-PP2A-KO mice. **a** Representative image of podocytes seeded on plates coated with fibronectin from Pod-PP2A-KO mice, monitored by live cell imaging for 30, 60 and 120 min. A modest but significant increase in cell spreading was observed in the isolated podocytes seeded on plates coated with fibronectin from Pod-PP2A–KO mice when compared with control. **b** Quantification of cell area was performed in each experiment. *n* = 3 experiments. **c** Primary podocytes were stained with a-tubulin and FITC labeled phalloidin to visualize the microtubule and F-actin cytoskeleton in physiological state, and no significant differences was observed. **d** Representative image of actin fiber in the primary podocyte after incubation with cytochalasin D for 30 min. The expression of actin fiber was significant down-regulated in primary podocytes after cytochalasin D treatment from Pod-PP2A-KO mice than that of control

To analyze the effects of PP2A on the function of cell spreading and migration, primary podocytes isolated from the control and Pod-PP2A–KO mice were seeded on plates coated with fibronectin, laminin or collagen type I. A modest but significant increase in cell spreading was observed in the isolated podocytes plated on fibronectin from Pod-PP2A–KO mice when compared with control, but not to laminin or collagen type I (Fig. 6a, Additional file 1: Figure S1, and quantified in Fig. 6b).

Horinouchi T, et al. revealed that inhibition of PP2A with okadaic acid in a conditionally immortalized mouse podocyte cell line suppressed microtubule elongation and abolished process formation [24]. However, no significant differences in the expression of actin fiber and α-tubulin (Fig. 6c) were observed between control and mutant podocytes in physiological state. To demonstrate the difference further, cytochalasin D was incubated with primary podocyte for 30 min to induce actin fiber disruption. Then actin fiber was greatly impaired in mutant podocytes when compared to the control (Fig. 6d). These results demonstrated that deletion of PP2A in podocyte impairs cytoskeleton rearrangement.

### Y-box binding protein-1 might be the target protein for dephosphorylation by PP2A in podocyte

**Fig. 7:**
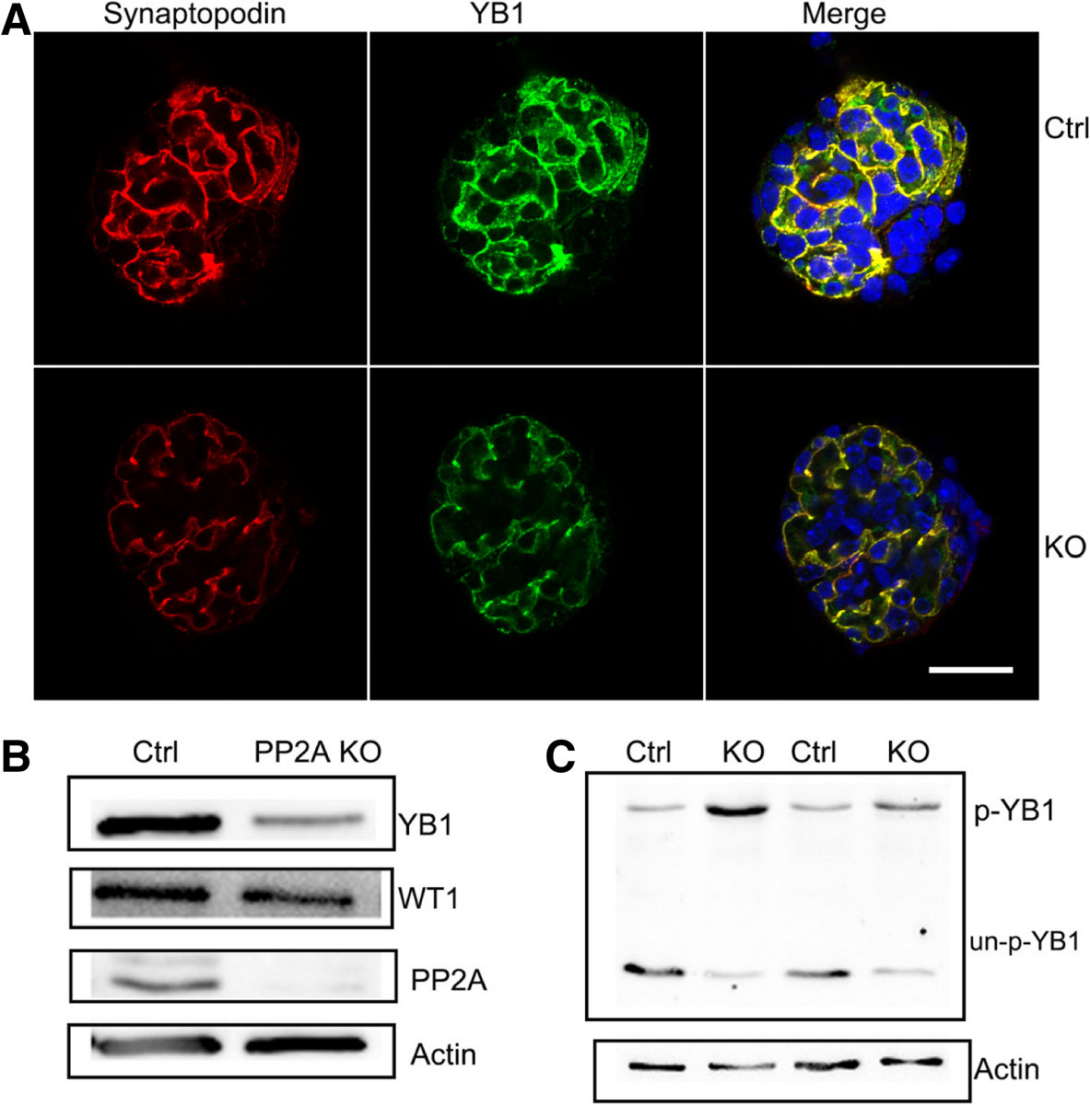
The expression pattern and variation of Y-box binding protein-1 (YB-1) in glomerular podocytes were studied by immunofluorescences and Western blot. **a** Representative confocal microscopic images of expression of YB-1 and synaptopodin in glomeruli from Pod-PP2A-KO mice and control. Scale bar: 50 μm. **b** The expression of total YB-1 was also down-regulated in podocytes from Pod-PP2A-KO mice by western blotting. **c** The phosphorylation of YB-1 (p-YB-1) is up-regulated, while un-phosphorylated YB-1 (un-p-YB-1) is down-regulated simultaneously in the podocytes from Pod-PP2A-KO mice by phostag

As loss of PP2A in podocyte leads to proteinuria and glomerulopathy, to gain further insight into its molecular mechanisms, primary podocyte from the Pod-PP2A-KO mice and the control were cultured, employing label-free nano-LC − MS/MS technology on a Q-exactive followed by SIEVE processing, then, the one of the most interesting protein is Y-box binding protein-1 (YB-1). The highly conserved YB-1 belongs to the family of cold-shock proteins that is of particular relevance in situations of cellular stress responses and mediates the renal fibrosis [26]. In present study, the expression pattern of YB-1 was studied in normal human kidney sections by immunofluorescences, which showed a podocyte−like staining pattern of YB-1 as synaptopodin. Meanwhile, the expression of YB-1 seems slightly down-regulated in kidney section by immunofluorescences from PP2A-KO mice, compared with the control mice as synaptopodin at 4 weeks of age (Fig. 7a). The expression level of total YB-1 (phosphorylation of YB-1 & un-phosphorylated YB-1 together) was also down-regulated in Pod-PP2A-KO mice by western blot (Fig. 7b).

YB-1 is phosphorylated at serine residue 174 which is up-regulated. Because Anti-YB-1 antibody to serine residue 102 was the only antibody to be found, so Phos-tag™ acrylamide was employed to detect the phosphorylation of YB-1, and the results showed that the phosphorylation of YB-1 (p-YB-1) was up-regulated, while un-phosphorylated YB-1 was down-regulated (un-p-YB-1) in the podocyte (Fig. 7c) from the Pod-PP2A-KO mouse compared to the controls.

## Discussion

Podocytes are fundamental and important component of the glomerular filtration barrier which functions to retain plasma protein in circulation [27]. The most striking feature of the podocyte is its ability to form the slit diaphragm [27]. Junctional proteins, as nephrin [28], podocin [29], ZO-1 [30] and CD2AP, are important determinant of the structural integrity of the slit diagram [29]. The congenital or acquired impairment of podocyte leads to loss of podocyte specific markers, process effacement and eventual detachment, and then results in proteinuria. Podocyte injury has been reported to be associated with several kidney diseases such as diabetic nephropathy, IgA nephropathy, focal segmental sclerosis and membranous glomerulonephritis [31]. Therefore, therapies aimed to preventing podocyte injury have major potentially clinical and economic benefits.

The vast majority of biological processes in eukaryotic cells are regulated by the balance between the kinases and phosphatases. Several studies have been done on the significance of kinases in kidney disease for decades, however, the roles of phosphatases remain poorly understood. As we already known, protein phosphatase 2A (PP2A) is the main serine/threonine phosphatase which function in the kidney [32]. While few previous studies focused on the expression, distribution of PP2A and its effects of cytoskeleton metabolism of podocytes, the role of PP2A in physiological function and mechanism for damage repairing remain unclear, moreover, therapies aimed at preventing podocyte injury is still a great challenge.

In this study, a conditional podocyte-specific PP2A knock-out mice were generated, and a series of podocyte and glomerular abnormalities were found, which included foot process effacement, glomerular proteinuria and progressive glomerulosclerosis. The data presented here may offer an alternative explanation for the role of PP2A in podocytes. We routinely observe proteinuria as early as at 5 weeks of age in the mutant mice. The elucidation of various hereditary glomerular diseases has revealed that proteinuria results from defects in the actin cytoskeleton, the slit diaphragm, the glomerular basement membrane, and podocyte loss. Interestingly in present study, deletion of PP2A in podocytes leads to the cytoskeleton rearrangement and down-regulation of synaptopodin, podocin, and nephrin but not loss of podocyte. Thus, our study not only demonstrated that PP2A as a critical component for the development of the maintenance of podocytes, but also suggested a potential role for PP2A as a therapeutic target in proteinuric kidney disease. Furthermore, in the spreading assay, a modest increase in cell spreading was observed in the isolated mutant podocytes plated on fibronectin when compared with control but not to laminin or collagen type I, however, the molecular mechanisms that mediate podocyte spreading is still unknown. We guess that PP2A may function in this behavior but it mechanism need to be elucidated further.

Label-free comparative proteomics was employed to probe the molecular mechanism of PP2A in maintaining podocyte function and preventing plasma protein leakage, and phosphorylation of YB-1 were found to be up-regulated in podocytes from Pod-PP2A-KO.

The highly conserved cold-shock protein YB-1 is a transcriptional and translational factor, which regulates many cellular processes such as cell proliferation, DNA repair, cellular stress response, cell differentiation, embryonic development and systemic & local (renal) inflammatory response [33–35]. Bergmann et al. [36] showed in a novel transgenic mouse model demonstrating human hemagglutinin tagged YB-1 provokes remarkably diverse breast carcinomas, and YB-1 knock-out mice are embryonic lethal due to neuronal defects reported by Uchiumi et al. [37].

The function of YB-1 in kidney remains to be elucidated. Previously, YB-1 was found to be expressed predominately in renal proximal tubular cells [38] and mesangial cells [26]. Dong et al. [39] uncovered increased blood serine protease activated protein C and associated stabilization of YB-1 protein protected the kidney from ischemia and reperfusion injury, which revealed an important role for YB-1 stabilization in nephron protection following acute kidney injury.

Alidousty et al. [40] examined YB-1 phosphorylation in the presence of the calcineurin inhibitor (CNI) (Cyclosporine A, CsA) in BL/6 mice. In contrast to samples from control animals that received only the solvent, the content of phosphorylated YB-1 (p-YB-1) and total YB-1 were strongly elevated in the nuclear compartment of kidney protein extracts following CsA challenge, and their findings point to a critical role of YB-1 in the resolution of inflammatory processes which may largely be due to calcineurin mediated dephosphorylation. Further, Wan et al. [41] reported that, YB-1 +/− animals displayed markedly reduced tubular injury, immune cell infiltration and renal fibrosis following ureteral obstruction, and, the therapeutic forced nuclear compartmentalization of phosphorylated YB-1 by the small molecule HSc025 attenuated fibrosis. Their results implied that phosphorylation and subcellular re-localization of YB-1 determined its effect on renal fibrosis. Recently, Gibbert L et al. [42] corroborated the pro-fibrotic role of YB-1 in glomeruli of patients under CNI-treatment. Such effects in glomeruli are significantly mitigated in CNI-treated mice with half-normal YB-1 expression (*YB-1+/−*). Surprisingly, in the tubulointerstitium we observe an opposite role of the CNI-YB-1 axis. Here, YB-1 is predominantly located to the nuclei and represses transcription of several extracellular matrix genes. Consistently, CNI-treatment in *YB-1+/−* mice markedly increases pro-fibrotic changes in the tubulointerstitium. In summary, our data provide evidence that fibrotic CNI-induced YB-1 effects in glomerular cells need to be contrasted with beneficial anti-fibrotic effects in the tubulointerstitium.

In the present study, YB-1 was found to be expressed in glomerular podocytes as synaptopodin-like pattern in the Pod-PP2A-KO mouse and control. Then YB-1 was identified to be the candidate target molecule for dephosphorylation effect of PP2A in glomerular podocytes by label-free nano-LC − MS/MS technology on a Q-exactive followed by SIEVE processing. Further, to confirm the result of label-free nano-LC − MS/MS technology, Phos-tag™ acrylamide was used to detect the expression of phosphorylation of YB-1 (p-YB-1) in the podocyte from the Pod-PP2A-KO mouse, and the phosphorylation of YB-1 was up-regulated in contrast to the wild type control. Furthermore, contrasted to the elevated expression of p-YB-1, total YB-1 (phosphorylated & un-phosphorylated YB-1 together) was moderately down-regulated in podocytes from the Pod-PP2A-KO mice detected by western blot and immunofluorescent staining. These results elucidated that conditional podocyte knockout of PP2A results in the up-regulation of p-YB-1 but down-regulation of un-phosphorylated YB-1 simultaneously. Our data may suggest that fine-tuning of YB-1 via a post-translational modification (balance between phosphorylation and dephosphorylation) by PP2A regulating its activity might be crucial for the functional integrity of podocytes, other than renal tubular epithelial cells and mesangial cells.

## Conclusion

In conclusion, our present study revealed the decreased expression of PP2A lead to reduced expression of slit diaphragm molecule and cytoskeleton rearrangement of podocytes. The mutant mice manifest a sequelae of podocyte and glomerular abnormalities. Meanwhile, fine-tuning of YB-1 via a post-translational modification by PP2A regulating its activity might be crucial for the functional integrity of podocytes and glomerular filtration barrier.

## Additional file

Additional file 1: **Figure S1.** Representative image of podocytes seeded on plates coated with collagen type I and laminin from PP2A-KO mice, monitored by live cell imaging for 30, 60 and 120 minutes. No obvious differences in cell spreading were observed from PP2A–KO mice when compared with control. (DOCX 570 kb)

## Data Availability

I can confirm I have included a statement regarding data and material availability in the declaration section of my manuscript.

## Abbreviations

ADM: Adriamycin
BSA: Bovine serum albumin
CsA: Cyclosporine A
EM: Electromicroscope
FP: Foot process
KO: Knock out
MPC5: Mouse podocyte cell line
PFA: Paraformaldehyde
Pod-PP2A–KO: Podocyte specific knockout of PP2A
PP2A: Protein phosphatase 2A
p-YB-1: Phosphorylation of YB-1
un-p-YB-1: Un-phosphorylated YB-1
YB-1: Y-box binding protein-1
