# Supplementary material for: Protein phosphatase 2A modulates podocyte maturation and glomerular functional integrity in mice
Source: Cell Commun Signal. 2019 Aug 6;17:91. doi: 10.1186/s12964-019-0402-y (PMC6685276; doi:10.1186/s12964-019-0402-y)
Supplement: Supplementary file 1 — Figure S1. Representative image of podocytes seeded on plates coated with collagen type I and laminin from PP2A-KO mice, monitored by live cell imaging for 30, 60 and 120 minutes. No obvious differences in cell spreading were observed from PP2A–KO mice when compared with control. (DOCX 570 kb) [file 12964_2019_402_MOESM1_ESM.docx]

**
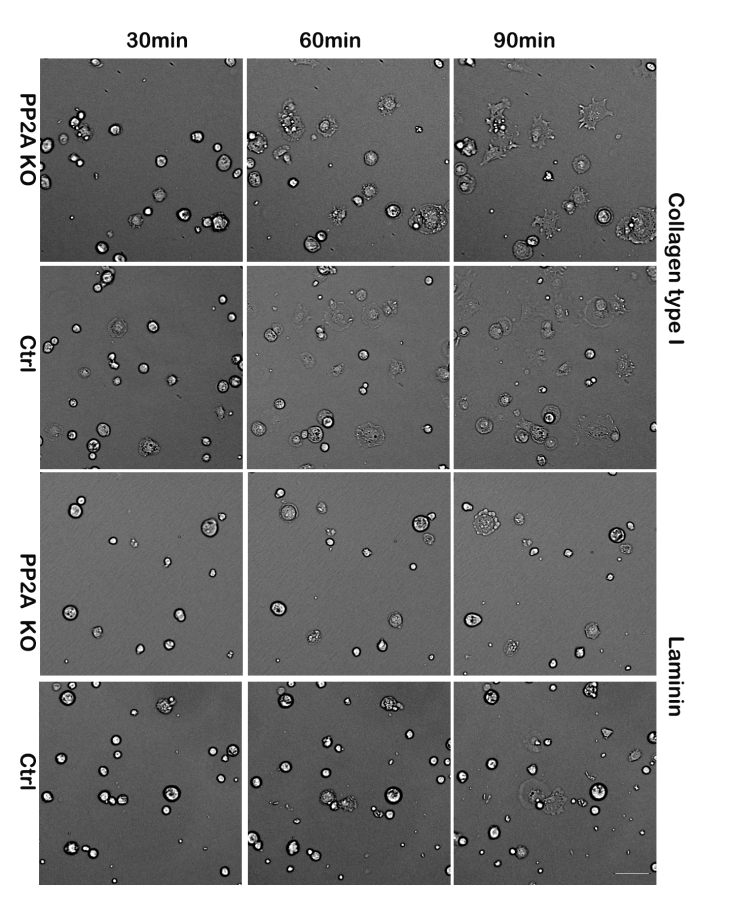
**

**Suplemental Figure 1**. Representative image of podocytes seeded on plates coated with collagen type I and laminin from PP2A-KO mice, monitored by live cell imaging for 30, 60 and 120 minutes. No obvious differences in cell spreading were observed from PP2A–KO mice when compared with control.
